# Salivary Biomarkers for Detection of Systemic Diseases

**DOI:** 10.1371/journal.pone.0061356

**Published:** 2013-04-24

**Authors:** Nilminie Rathnayake, Sigvard Åkerman, Björn Klinge, Nina Lundegren, Henrik Jansson, Ylva Tryselius, Timo Sorsa, Anders Gustafsson

**Affiliations:** 1 Karolinska Institutet, Department of Dental Medicine, Division of Periodontology, Stockholm, Sweden; 2 Malmö University, Faculty of Odontology, Department of Oral Diagnostics, Malmö, Sweden; 3 Malmö University, Faculty of Odontology, Department of Periodontology, Malmö, Sweden; 4 University of Helsinki, Helsinki University Central Hospital, Department of Oral and Maxillofacial Diseases, Helsinki, Finland; National Institute for Infectious Diseases (L. Spallanzani), Italy

## Abstract

**Background and Objective:**

Analysis of inflammatory biomarkers in saliva could offer an attractive opportunity for the diagnosis of different systemic conditions specifically in epidemiological surveys. The aim of this study was to investigate if certain salivary biomarkers could be used for detection of common systemic diseases.

**Materials and Methods:**

A randomly selected sample of 1000 adults living in Skåne, a county in the southern part of Sweden, was invited to participate in a clinical study of oral health. 451 individuals were enrolled in this investigation, 51% women. All participants were asked to fill out a questionnaire, history was taken, a clinical examination was made and stimulated saliva samples were collected. Salivary concentrations of IL-1β, -6, -8, TNF-α, lysozyme, MMP-8 and TIMP-1 were determined using ELISA, IFMA or Luminex assays.

**Results:**

Salivary IL-8 concentration was found to be twice as high in subjects who had experience of tumour diseases. In addition, IL-8 levels were also elevated in patients with bowel disease. MMP-8 levels were elevated in saliva from patients after cardiac surgery or suffering from diabetes, and muscle and joint diseases. The levels of IL-1β, IL-8 and MMP-8, as well as the MMP-8/TIMP-1 ratio were higher in subjects with muscle and joint diseases.

**Conclusion:**

Biomarkers in saliva have the potential to be used for screening purposes in epidemiological studies. The relatively unspecific inflammatory markers used in this study can not be used for diagnosis of specific diseases but can be seen as markers for increased systemic inflammation.

## Introduction

Analysis of inflammatory biomarkers in saliva could offer an attractive solution for the diagnosis of different systemic conditions in epidemiological surveys.

Human saliva is composed of 98% water and 2% other compounds, such as electrolytes, mucus, antibacterial compounds, and various enzymes. Multiple functions of saliva include rinsing, solubilisation of food substances, food and bacterial clearance, lubrication of soft tissues, bolus formation, swallowing, speech and facilitation of mastication, all of which are related to its fluid characteristics and specific components. In addition, saliva components contribute to mucosal coating, digestion and antibacterial defence [1].

Several types of inflammatory biomarkers associated with both oral diseases, as well as systemic diseases have been detected in saliva, such as interleukins-1β, -6 and -8 (IL-1β, -6 and -8), tumour necrosis factor-α (TNF-α) and matrix metalloproteinases (MMP)-8 and -9 [2], [3], [4], [5], [6]. An increasing number of specific molecular markers for different diseases, such as oral and breast cancer, cardiovascular diseases and human immunodeficiency virus (HIV) are being identified [7], [8], [9].

IL-1β is a pro-inflammatory cytokine released by different cells in the inflammatory lesion, and IL-6 has both pro-inflammatory and anti-inflammatory actions [10], [11]. It can induce B and T- cell growth and differentiation, and acute- phase protein induction. IL-8 is a chemokine, mainly produced by macrophages and epithelial cells upon inflammatory stimulation. IL-8 plays a causative role in the acute inflammatory phase by activating neutrophils and thus has an important role in the inflammatory response [12], [13]. TNF-α plays a vital role as a pro-inflammatory and immune-regulatory cytokine [14]. MMP-8 or collagenase-2 derived mainly from polymorphonuclear leukocytes (PMN) is particularly released during the acute phase of inflammatory diseases [15]. It is known that MMP-8 is involved in the pathogenesis of coronary artery diseases. Activation of MMPs plays an important role in infarction evolution as well as in tissue repairing processes and cardiac remodelling [16], [17]. As a result of acute myocardial infarction, a reparative process is initiated by infiltration of inflammatory cells, MMP activation, remodelling of extracellular matrix and scar formation [18]. MMP-8 and tissue inhibitor of MMP (TIMP) -1 concentrations have been associated with ischemia and infarction [19]. The initiation of collagen degradation from the connective tissue and alveolar bone occurs due to an imbalance between MMPs and their inhibitors TIMPs [20]. TIMP-1 is a major endogenous inhibitor of MMP-8. MMP-8 and TIMP-1 levels, as well as the MMP-8/TIMP-1 ratio in serum reflect progression and severity of cardiovascular diseases [21], [22]. Additional enzymes that are involved have been identified in saliva include lysozymes, which are also abundant in a number of other secretions, such as tears and mucus. Lysozymes are glycoside hydrolases and part of the innate immune system. Furthermore, one of the main properties of lysozymes is their ability to bind to hydroxyapatite, which suggests an antimicrobial role on the tooth surface [23].

IL-1β, MMP-8 and TNF-α have been shown to be elevated in the inflamed joints and serum of patients with rheumatoid arthritis [24]. Another study showed that the salivary levels of IL-1β which was the only biomarker, was significantly higher in the arthritis group compared to the control group [25].

With this background we wish to test the hypothesis that certain salivary biomarkers could be used for the detection of systemic diseases, with particular relevance to epidemiological studies. Special attention was addressed to the effects of smoking on potential salivary inflammatory biomarkers.

## Materials and Methods

### Participants

A randomly selected sample of 1000 adults living in Skåne, a county in the southern part of Sweden, was invited to participate in a clinical study of oral health. The individuals were between 20 and 89 years old and were registered living in Skåne during 2007. The participant list was obtained from SPAR (The Swedish Government’s Person Address Register) and included background variables, such as gender, domicile, address and age in 5-year-intervals. All participants gave their written informed consent to the study protocol, which had been approved by the Ethical Board (Dnr 513/2006) at the University of Lund, Sweden. Of the original sample, 11 individuals had moved from the region, 14 had an unknown address and nine were deceased, thus leaving 966 individuals as the initial sample. In total, 448 individuals were examined clinically, 230 (51%) women and 218 (49%) men (Figure S1 in File S1). The clinical examinations were performed between March 2007 and November 2008, and took place at the Faculty of Odontology at Malmö University, Sweden, and at three clinics belonging to the Public Dental Service in Helsingborg, Kristianstad and Ystad, all in Skåne [26].

### Questionnaire

All the clinically examined participants were asked to respond to a questionnaire, and only one failed to answer. This questionnaire contained 58 questions concerning patient perception of oral health, oral healthcare needs, pain, the use of oral healthcare, dental materials and background factors [27]. Anamnestic data, including diseases, use of medication, smoking and the use of Swedish snus were collected. Patients who were not able to or were not interested in being clinically examined were contacted by telephone in order to be part of a non-response analysis, and 175 of these individuals answered the same questionnaire as the individuals participating in the clinical study. Individuals who filled in the questionnaire as a non-response analysis were more likely to be born in Sweden, have a lower educational level and were missing a higher number of teeth than those participating in the clinical study. These differences can most likely be explained by the fact that the non-response group included most of the oldest individuals compared to the clinical study. The individuals, who neither participated in the clinical examination nor answered the questionnaire, were those who could not be reached by telephone or letter, were unable to take part due to bad health, due to old age or were simply not interested in participating in this study.

### Salivary Sampling

Stimulated saliva was collected during 5 min chewing on 0.5 g of paraffin, into a graded test-tube. The collected amount was determined, excluding the foam, and the secretion rate per minute was recorded. Trained dental assistants performed saliva sampling and scoring of results. Collected samples were immediately frozen at −20°C until processing. The saliva samples were collected in 15 ml Falcon tubes (kolla artikelnumret!) and kept frozen at −20°C until further processed. The samples were then thawed and centrifuged at 500 g for 10 minutes at 4°C. The supernatants were collected and divided into several aliquots that were quick-frozen and stored at −80°C until analysis. Each aliquot was used only once in an assay, and then discarded. The pellets, containing whole cells, were resuspended in 200 ul PBS and transferred to a 1.5 ml Eppendorf tube (Eppendorf, Hauppauge, NY, USA), and immediately frozen and stored at −80°C.

### Biomarker Analysis

MMP-8 was analysed by a time-resolved immunofluorometric assay. Monoclonal MMP-8- antibodies 8708 and 8706 (Medix Biochemica, Kauniainen, Finland) were used as capture antibodies and tracer antibodies respectively. The tracer antibody (8706) was labelled using europium (Eu)-chelate and the assay buffer contained 20 mM Tris-HCl (pH 7.5), 0.5 M NaCl, 5 mM CaCl2 50 lM ZnCl2, 0.5% bovine serum albumin, 0.05% sodium azide and 20 mg/l diethylenetriaminepentaacetic acid. Saliva samples were diluted in assay buffer and incubated for 1 h, followed by incubation for a further 1 h with tracer antibody. Enhancement solution was added, and after 5 min, fluorescence was measured using a 1234 Delfia Research Fluorometer (Wallac, Turku, Finland) [20], [28]. The concentration of capture antibody (8708) in the MMP-8/IFMA assay was 2.25 ug/well, and the concentration of the tracer Eu-lablled MMP-8 antibody (8706) was 0.5 ug/well. MMP-8 had a detection limit of 0.08 ng/ml and a coefficient of variation of 7.3%. TIMP-1 (Amersham Biotrak, GE Healthcare, Buckinghamshire, UK) was analysed by an enzyme-linked immunosorbent assay (ELISA) kits according to the manufacturer’s instructions. The inter-assay coefficient of variation for TIMP-1 was 8.2% and the detection limit for the assay was 1.25 ng/ml. Concentrations of salivary TNF-α, IL-1β, IL-6 and IL-8 were determined by using a Luminex multiplex kit (BioRAD, Hercules, CA, USA). Lysozyme was analysed with ELISA kit (R&D Systems, Minneapolis, MN, USA). The detection limits for analysed cytokines were as follows: TNF-α 1.2 pg/ml, IL-1β 0.5 pg/ml, IL-6 0.5 pg/ml and IL-8 0.5 pg/ml and for lysozyme 1.25 ng/ml. Sample analyses were performed at the Department of Dental Medicine, Karolinska Institutet. The total protein concentration was determined using the Bradford assay. The amounts of the analysed substances were expressed as ng or pg per ml of centrifuged saliva.

### Statistical Analysis

Data analyses were performed using Statistical Package for Social Sciences (SPSS) version 21 (SPSS Inc., Chicago, IL). The p-value ≤0.05 was considered as significant. The significances of the differences in biomarker concentration between smoker/non-smoker, men/women and between those suffering with or without the indicated disease were calculated with Students t-test. The significance of the differences after compensation for age, gender and smoking was performed using the General Linear Model. The correlation between age and biomarker concentration was calculated with Pearson Product Moment Correlation. Values under the detection limit were not included into the statistical analysis.

## Results

The characteristics of the study population (age, smoking, and systemic conditions) are presented in Table S1 in File S2. The mean age was higher in the male subjects. The percentage of tobacco smoking, heart surgery, heart disease, hypertension and bowel diseases were also higher in males. In female subjects, a number of conditions were found to be more prevalent, for example diabetes, muscle and joint diseases, tumours and mental disorders.

The analysed biomarkers were detected in more than 99% of the samples, except for TNF-α whose concentration was below the detection level in more than 50% of the samples. Table S2 in File S2 shows the influence of gender, smoking and age on the concentration of analysed inflammatory biomarkers (IL-1β, -6, -8, TNF-α, lysozyme, MMP-8, TIMP-1 and total protein concentration). Salivary concentrations of IL-1β, and IL-8 were higher in male subjects compared to the female subjects or otherwise there were no gender related differences. Furthermore, the levels of IL-8, MMP-8, the ratio of MMP-8/TIMP-1 and the total protein concentration were higher in smokers. Finally, all the analysed biomarkers, except IL-6 and TIMP-1, correlated significantly with the variable age.

Tables S3a, S3b and S3c in File S3 illustrate mean concentrations and standard deviations of analysed biomarkers in stimulated saliva samples from 441 subjects. The analysed biomarkers did not differ between those who answered that they had heart disease and those who did not. However, the 11 patients that had undergone heart surgery had higher concentrations of MMP-8. This difference also remained after compensation for age, gender and smoking habits. Patients reporting high blood pressure presented with higher concentrations of MMP-8 and lysozyme, as well as an increased MMP-8/TIMP-1 ratio but these differences were lost after compensation for age, gender and smoking habits.

Patients with diabetes had higher concentrations of MMP-8 and a higher MMP-8/TIMP-1 ratio. These differences were more pronounced after compensation for age, gender and smoking. Bowel disease was associated with elevated concentrations of IL-8 and total protein. Muscle and joint diseases were associated with increased IL-1β, MMP-8 and the ratio MMP-8/TIMP-1. Patients who responded that they were or had been suffering from any kind of tumour had more than twice the concentration of IL-8 compared to those who had not had tumours. This difference was significant and remained after compensation. Mental illness could not be associated with any of the analysed biomarkers.

Variable inflammation, which could include a combination of systemic conditions like heart disease, high blood pressure, bowel disease, and muscle and joint diseases, was associated with increased concentrations of IL-8, MMP-8, MMP-8/TIMP-1 ratio, lysozyme and total protein. Out of the above mentioned conditions only the total protein concentration difference remained significant after compensation for age, gender and smoking.

## Discussion

Saliva is a unique fluid that can be used to monitor both oral and systemic health. In the present study, salivary levels of IL-1β, -6 -8, TNF-α, MMP-8, TIMP-1 and lysozyme were determined in a population of 441 adults. It shows that several common diseases or conditions can be associated with the studied biomarkers in saliva, supporting our hypothesis. Indeed, the numerically recovered biggest differences between patients with or without the studied diseases were specifically found for diabetes and tumour diseases.

Patients with diabetes had a three times higher ratio of MMP-8/TIMP-1 and twice as high concentration of MMP-8. Increased MMP-8 levels in association with diabetes have earlier been shown in saliva, gingival crevicular fluid and in gingival tissues [29], [30], [31]. Increased concentrations of MMP-8 have been associated with an increased inflammatory process in patients with diabetes [32]. Salivary IL-8 concentration was found to be twice as high in patients with experience of tumour diseases compared to subjects who had not suffered. This difference was significant even after compensation for differences in age, gender and smoking habits. Our finding is in line with a study performed by Wei and co-authors that investigated salivary IL-8 levels of patients with oral cancer [33]. A recent study by Punyani and Sathawane [34] suggested that salivary IL-8 had the potential to be biomarker for oral squamous cell carcinoma (OSCC). An earlier study using transcriptome analyses also showed 91% sensitivity for IL-8 as a marker for OSCC [35]. In addition, IL-8 has been shown to be elevated in both serum and saliva in patients with head and neck squamous cell cancer [36]. Interestingly, in the current study, IL-8 was also raised in patients who reported bowel disease, and muscle and joint diseases.

In recent years a possible role for MMPs in atherosclerosis development has been described [37]. MMP-8 is reported to be associated with both atherosclerosis and metabolic syndrome [38], [39], [40]. Elevated salivary levels of MMP-8 have been linked with an increased risk for cardiovascular disease [41]. In our study, participants who responded that they had heart disease did not have elevated concentrations of MMP-8. Nevertheless, those who had underwent heart surgery (n = 11) had significantly higher MMP-8 concentrations in their saliva.

In earlier publications, oral infections, hyperglycemia, hypertension and metabolic syndrome show a significant association with increased salivary levels of lysozyme [42], [43]. In our investigation, lysozyme concentration was higher in the saliva of patients reporting inflammatory conditions and/or high blood pressure. The significance of these associations disappeared after compensation for differences in gender, age and smoking habits between the groups.

Oral infections, such as periodontal disease could have also influenced our findings as periodontal disease has been associated with conditions like cardiovascular disease, diabetes mellitus and rheumatoid arthritis [44], [45], [46]. Salivary biomarkers such as IL-1β, MMP-8 and the ratio of MMP-8/TIMP-1 correlated significantly with clinical variables like pocket depth and bleeding on probing, which could reflect the degree of periodontal/gingival inflammation [47]. Further, IL-1β could be seen as a general marker of inflammation but it remains to be determined if it is possible to differentiate between gingivitis and periodontitis. Higher salivary concentration of IL-1β from patients with periodontitis has been detected in several earlier studies [48], 49,50. A study performed by Kinney et al. reported that IL-1β and MMP-8 strongly correlated with periodontal disease status [51].

In this study we chose to relate the amounts of the analysed proteins to the volume rather than to the protein content. Our study indicates that certain biomarkers in saliva exhibit a potential to be used for screening purposes in epidemiological studies. Conversely, it is important to consider that both IL-1β and MMP-8 are general markers for both local and systemic inflammations and seemingly cannot be used for diagnosis of specific diseases. The mean value of MMP-8 was more than 50% higher in patients with one or several inflammatory conditions (heart disease, high blood pressure, bowel disease, and muscle and joint diseases). Additional investigations are warranted to develop tools for diagnosis and intervention selection, as well as for screening purposes [30].

This study has some limitations that must be considered when interpreting the findings: the medical status is based on the participantś self-assessment and no verification of the answers from the anamnestic data. There is an obvious risk for false negatives, for instance undiagnosed diseases. In addition, due to the large number of comparisons in regard to different systemic conditions, compensation for this will eliminate all significances. However, this study is meant as a survey to generate hypotheses.

Since the inflammatory markers analysed in this study are general, more specific markers would be required to achieve an acceptable specificity. A combination of markers was probably necessary for several diseases [52]. Furthermore, the biomarkers obtained from the saliva samples depend upon the biochemical nature of the marker, the source and type of sample being taken, the assay reagents and techniques used [41], [53], and the mechanism by which the marker enters the oral cavity [54].

In conclusion, saliva-based clinical testing could provide a potential diagnostic tool for the detection of certain diseases and conditions using biomarkers associated with enhanced systemic inflammation. Although limited by the study design, additional research in this area is required to strengthen this assessment.

## Supporting Information

File S1 **Figure S1: Flow-chart showing the selection of the participants.**
(DOCX)

File S2 **Table S1: Characterisation and clinical parameters of the study population & Table S2: The influence of age, smoking and gender on the analysed salivary biomarkers.**
(DOCX)

File S3 **Table S3a, S3b and S3c. Illustrates mean concentrations and standard deviations of the analysed salivary biomarkers of stimulated saliva samples.**
(DOCX)
