# Supplementary material for: Salivary Biomarkers for Detection of Systemic Diseases
Source: PLoS One. 2013 Apr 24;8(4):e61356. doi: 10.1371/journal.pone.0061356 (PMC3634781; doi:10.1371/journal.pone.0061356)
Supplement: File S1 — Figure S1: Flow-chart showing the selection of the participants. (DOCX) [file pone.0061356.s001.docx]

**Figure S1**

Excluded (N=34)

- Moved from the region (N=11)
- Unknown address (N=14)
- Deceased (N=9)

## Analysis

Saliva Samples

(N=441)

Assessed for eligibility

(N=1000)

Randomized

(N=966)

## Allocation

Women

(N=232)

Enrolled subjects with clinical examination (N=451)

Decline to participate

(N=515)

Men

(N=219)

## Enrollment
