# Supplementary material for: Salivary Biomarkers for Detection of Systemic Diseases
Source: PLoS One. 2013 Apr 24;8(4):e61356. doi: 10.1371/journal.pone.0061356 (PMC3634781; doi:10.1371/journal.pone.0061356)
Supplement: File S2 — Table S1: Characterisation and clinical parameters of the study population & Table S2: The influence of age, smoking and gender on the analysed salivary biomarkers. (DOCX) [file pone.0061356.s002.docx]

**Table S1in File S2.** Characteristics and clinical parameters of the study population (n=448)

| **Parameter** | **Female (n=230)** | **Male** **(n=218)** | **Total (n=448)** | *p* |
| --- | --- | --- | --- | --- |
| Age (mean±SD) | 47.9 ±17.1 | 49.3±16.9 | 48.6±17.0 | 0.390 |
| Smoking (%) | 16.1 | 17.8 | 16.9 | 0.627 |
| Heart surgery (%) | 1.3 | 3.7 | 2.5 | 0.107 |
| Heart disease (%) | 7.5 | 8.4 | 7.9 | 0.741 |
| Hypertention (%) | 14.2 | 20.5 | 7.2 | 0.080 |
| Diabetes (%) | 3.9 | 3.2 | 3.6 | 0.682 |
| Bowel diseases (%) | 6.6 | 7.4 | 7.0 | 0.741 |
| Muscle and joint diseases (%) | 25.7 | 20.5 | 23.1 | 0.196 |
| Tumor (%) | 4.0 | 3.3 | 3.6 | 0.683 |
| Mental disordes (%) | 8.8 | 2.8 | 5.9 | 0.007 |

**Table S2 in File S2**. The influence of gender, smoking, and age on the concentration of analysed biomarkers and the total protein concentration from stimulated saliva samples (n=441)

| **Biomarker** | **Gender** | | *p* | **Smoking** | | *p* | **Correlation with age** | |
| --- | --- | --- | --- | --- | --- | --- | --- | --- |
|  | **Female** | **Male** |  | **No** | **Yes** |  | R | *p* |
|  | **n=226** | **n=215** |  | **n=366** | **n=75** |  |  |  |
|  | **Mean±SD** | **Mean±SD** |  | **Mean±SD** | **Mean±SD** |  |  |  |
| IL-1β pg/ml | 62.7±117.0 | 87.2±114.8 | 0.027 | 79.3±122.9 | 52.2±74.8 | 0.066 | 0.129 | 0.003 |
| IL-6 pg/ml | 7.5±8.9 | 8.4±13.6 | 0.398 | 8.1±12.0 | 7.1±7.9 | 0.474 | 0.034 | 0.238 |
| IL-8 pg/ml | 411.8±445.7 | 601.3±898.5 | 0.005 | 537.4±758.3 | 342.1±351.4 | 0.030 | 0.164 | 0.001 |
| Lysozyme ng/ml | 362.1±359.5 | 431.1±484.6 | 0.089 | 391.5±413.3 | 416.4±485.6 | 0.645 | -0.214 | 0.001 |
| TNF-α pg/ml* | 5.9±15.4 | 4.1±7.7 | 0.114 | 4.0±8.3 | 10.1±23.2 | 0.001 | -0.163 | 0.001 |
| MMP-8 ng/ml | 287.3±235.2 | 301.1±295.6 | 0.587 | 305.3±271.3 | 239.2±233.4 | 0.050 | 0.183 | 0.001 |
| TIMP-1 ng/ml | 253.3±192.6 | 274.0±194.9 | 0.265 | 258.5±189.2 | 287.6±241.8 | 0.237 | -0.003 | 0.477 |
| MMP-8/ TIMP-1 | 0.58 ±0.76 | 0.56±0.95 | 0.891 | 0.60 ± 0.92 | 0.35±0.34 | 0.021 | 0.209 | 0.001 |
| Total protein ug/ml | 833.3±448.1 | 808.9±406.3 | 0.548 | 839.9±431.0 | 730.1±403.2 | 0.044 | 0.114 | 0.008 |

Statistical significance of the difference between male and female, and smoker and non-smokers were calculated with Students t-test. The correlation

to age was calculated with Pearson Product Moment Correlation

* TNF-α could be detected in less than half of the total samples
