# Supplementary material for: Salivary Biomarkers for Detection of Systemic Diseases
Source: PLoS One. 2013 Apr 24;8(4):e61356. doi: 10.1371/journal.pone.0061356 (PMC3634781; doi:10.1371/journal.pone.0061356)
Supplement: File S3 — Table S3a, S3b and S3c. Illustrates mean concentrations and standard deviations of the analysed salivary biomarkers of stimulated saliva samples. (DOCX) [file pone.0061356.s003.docx]

**Table S3a in File S3.** Concentrations (mean and SD) for analysed biomarkers in samples of stimulated saliva from 441 subjects.

| **Biomarker** | **Heart disease** | *p1* | *p2* | **Heart surgery** | *p1* | *p2* | **Hypertension** | *p1* | *p2* |
| --- | --- | --- | --- | --- | --- | --- | --- | --- | --- |
|  | **Yes - 35** |  |  | **Yes - 11** |  |  | **Yes - 76** |  |  |
|  | **No - 406** |  |  | **No - 430** |  |  | **No - 365** |  |  |
|  | **Mean±SD** |  |  | **Mean±SD** |  |  | **Mean±SD** |  |  |
| IL-1β(pg/ml) | 88.8±180.8 | 0.454 | 0.739 | 40.4±68.2 | 0.324 | 0.126 | 94.5 ±165.8 | 0.103 | 0.904 |
|  | 73.5±109.4 |  |  | 75.6±117.4 |  |  | 70.6±103.1 |  |  |
| IL-6(pg/ml) | 6.7±8.1 | 0.525 | 0.528 | 7.0±4.5 | 0.779 | 0.775 | 7.8±13.1 | 0.905 | 0.983 |
|  | 8.0±11.8 |  |  | 7.9±11.6 |  |  | 7.9±11.1 |  |  |
| IL-8(pg/ml) | 516.9±582.6 | 0.912 | 0.315 | 340.0±370.9 | 0.438 | 0.130 | 570.0±781.4 | 0.374 | 0.241 |
|  | 503.1±719.8 |  |  | 508.4±715.7 |  |  | 490.5±693.8 |  |  |
| MMP-8(ng/ml) | 373.8±355.7 | 0.065 | 0.573 | 472.9±570.3 | 0.024 | 0.040 | 370.6±386.1 | 0.006 | 0.414 |
|  | 287.2±256.4 |  |  | 289.5±253.4 |  |  | 278.1±231.2 |  |  |
| TIMP-1(ng/ml) | 231.6±153.9 | 0.126 | 0.265 | 289.2±209.8 | 0.656 | 0.671 | 238.0±176.6 | 0.208 | 0.132 |
|  | 266.2±196.8 |  |  | 262.8±193.6 |  |  | 268.8±197.0 |  |  |
| MMP-8/TIMP-1 | 0.76±1.24 | 0.143 | 0.977 | 0.55±0.77 | 0.972 | 0.758 | 0.78±1.16 | 0.012 | 0.828 |
|  | 0.54±0.81 |  |  | 0.56±0.86 |  |  | 0.51±0.77 |  |  |
| Lysozyme(ng/ml) | 373.4±462.3 | 0.748 | 0.411 | 522.3±745.4 | 0.319 | 0.126 | 307.3±325.4 | 0.047 | 0.566 |
|  | 397.6±423.3 |  |  | 392.5±415.6 |  |  | 414.1±442.2 |  |  |
| Total protein concentration(ug/ml) | 847.9±408.6 | 0.703 | 0.780 | 807.6±499.2 | 0.914 | 0.761 | 876.8±481.9 | 0.215 | 0.913 |
|  | 819.1±430.0 |  |  | 821.7±426.6 |  |  | 809.9±415.6 |  |  |

*p*1 indicates significance of the differences after a bivariate comparison

*p*2 indicates the significance after compensation for differences in gender, age and smoking habits.

**Table S3b in File S3.** Concentrations (mean and SD) for analysed biomarkers in samples of stimulated saliva from 441 subjects.

| **Biomarker** | **Diabetes** | *p1* | *p2* | **Bowel diseases** | *p1* | *p2* | **Muscle/joint diseases** | *p1* | *p2* |
| --- | --- | --- | --- | --- | --- | --- | --- | --- | --- |
|  | **Yes - 16** |  |  | **Yes - 31** |  |  | **Yes - 102** |  |  |
|  | **No - 425** |  |  | **No - 410** |  |  | **No - 339** |  |  |
|  | **Mean±SD** |  |  | **Mean±SD** |  |  | **Mean±SD** |  |  |
| IL-1β(pg/ml) | 65.0±80.6 | 0.636 | 0.399 | 87.5±103.6 | 0.526 | 0.712 | 101.2±154.1 | 0.008 | 0.017 |
|  | 75.0±117.7 |  |  | 73.7±117.5 |  |  | 66.7±101.5 |  |  |
| IL-6(pg/ml) | 8.2±6.1 | 0.930 | 0.805 | 8.9±10.2 | 0.603 | 0.471 | 7.9±11.2 | 1.000 | 0.686 |
|  | 7.9±11.6 |  |  | 7.8±11.5 |  |  | 7.9±11.5 |  |  |
| IL-8(pg/ml) | 583.8±538.0 | 0.876 | 0.872 | 922.2±1598.3 | 0.001 | 0.008 | 634.3±866.2 | 0.034 | 0.159 |
|  | 501.2±715.3 |  |  | 472.6±582.8 |  |  | 465.1±651.3 |  |  |
| MMP-8(ng/ml) | 480.6±402.3 | 0.075 | 0.021 | 297.1±216.6 | 0.948 | 0.799 | 356.3±331.2 | 0.007 | 0.035 |
|  | 287.0±257.7 |  |  | 293.8±269.8 |  |  | 275.3±240.7 |  |  |
| TIMP-1(ng/ml) | 193.9±209.9 | 0.195 | 0.152 | 286.0±231.2 | 0.503 | 0.461 | 279.5±213.0 | 0.341 | 0.262 |
|  | 266.1±192.9 |  |  | 261.8±190.9 |  |  | 258.6±187.7 |  |  |
| MMP-8/TIMP-1 | 1.76±2.45 | 0.060 | 0.001 | 0.44±0.39 | 0.411 | 0.160 | 0.77±1.40 | 0.005 | 0.047 |
|  | 0.52±0.70 |  |  | 0.57±0.88 |  |  | 0.50±0.59 |  |  |
| Lysozyme(ng/ml) | 337.6±172.3 | 0.221 | 0.747 | 451.5±516.4 | 0.451 | 0.174 | 336.7±264.9 | 0.111 | 0.949 |
|  | 397.9±432.6 |  |  | 391.5±418.8 |  |  | 413.5±462.5 |  |  |
| Total protein concentration(ug/ml) | 866.8±235.3 | 0.459 | 0.958 | 1044.4±715.1 | 0.003 | 0.004 | 900.5 ± 491.0 | 0.033 | 0.113 |
|  | 819.7±433.6 |  |  | 804.5±394.2 |  |  | 797.6 ± 405.8 |  |  |

*p*1 indicates significance of the differences after a bivariate comparison

*p*2 indicates the significance after compensation for differences in gender, age and smoking habits.

**Table S3c in File S3.** Concentrations (mean and SD) for analysed biomarkers in samples of stimulated saliva from 441 subjects.

| **Biomarker** | **Tumour** | *p 1* | *p 2* | **Mental illness** | *p 1* | *p 2* | **Inflammation** | *p 1* | *p 2* |
| --- | --- | --- | --- | --- | --- | --- | --- | --- | --- |
|  | **Yes - 16** |  |  | **Yes - 26** |  |  | **Yes - 181** |  |  |
|  | **No - 425** |  |  | **No - 415** |  |  | **No - 260** |  |  |
|  | **Mean±SD** |  |  | **Mean±SD** |  |  | **Mean±SD** |  |  |
| IL-1β(pg/ml) | 133.8±199.3 | 0.038 | 0.204 | 62.3±103.9 | 0.576 | 0.839 | 84.5±129.0 | 0.142 | 0.873 |
|  | 72.4±112.0 |  |  | 75.5±117.3 |  |  | 67.9±106.8 |  |  |
| IL-6(pg/ml) | 12.8±20.7 | 0.081 | 0.077 | 5.1±5.6 | 0.196 | 0.243 | 7.9±11.0 | 0.966 | 0.593 |
|  | 7.7±10.9 |  |  | 8.1±11.7 |  |  | 7.9±11.7 |  |  |
| IL-8(pg/ml) | 1137.4±1539.6 | 0.001 | 0.005 | 352.2±336.7 | 0.261 | 0.439 | 605.5±901.3 | 0.013 | 0.478 |
|  | 480.4±650.1 |  |  | 513.7±725.3 |  |  | 435.0±531.5 |  |  |
| MMP-8(ng/ml) | 348.1±300.2 | 0.409 | 0.886 | 235.3±218.5 | 0.247 | 0.244 | 344.0±314.6 | 0.001 | 0.070 |
|  | 292.0±265.0 |  |  | 297.7±268.7 |  |  | 260.0±221.5 |  |  |
| TIMP-1(ng/ml) | 295.9±207.0 | 0.497 | 0.318 | 277.4±220.6 | 0.707 | 0.708 | 273.1±208.5 | 0.388 | 0.310 |
|  | 262.3±193.4 |  |  | 262.6±192.3 |  |  | 256.9±183.2 |  |  |
| MMP-8/TIMP-1 | 0.61±0.85 | 0.820 | 0.483 | 0.42±0.49 | 0.396 | 0.320 | 0.68±1.13 | 0.015 | 0.673 |
|  | 0.56±0.86 |  |  | 0.57±0.87 |  |  | 0.48±0.59 |  |  |
| Lysozyme(ng/ml) | 412.3±357.7 | 0.874 | 0.242 | 405.8±543.1 | 0.901 | 0.687 | 345.9±352.2 | 0.042 | 0.901 |
|  | 395.1±428.7 |  |  | 395.1±418.4 |  |  | 429.7±467.4 |  |  |
| Total protein concentration(ug/ml) | 970.6±492.3 | 0.156 | 0.358 | 832.2±489.0 | 0.894 | 0.809 | 895.8±510.0 | 0.002 | 0.026 |
|  | 815.7±424.9 |  |  | 820.7±424.5 |  |  | 770.5±353.4 |  |  |

*p*1 indicates significance of the differences after a bivariate comparison

*p*2 indicates the significance after compensation for differences in gender, age and smoking habits.
